# Sociocultural Costs of the Long-term COVID-19 Outbreak in Bangladesh: A Systematic Review

**DOI:** 10.1177/21582440221143298

**Published:** 2022-12-20

**Authors:** Sanjoy Kumar Chanda, Md. Ripul Kabir, Tuhin Roy, Tunvir Ahamed Shohel, Md. Hasan Howlader, Shaharior Rahman Razu

**Affiliations:** 1 Khulna University, Khulna, Bangladesh

**Keywords:** sociocultural costs, COVID-19, education, social relationship, gender violence, Rohingya refugee, Bangladesh

## Abstract

Beyond the physical transmission of COVID-19, the pandemic has had far-reaching consequences in Bangladesh, including social and cultural implications. This review paper aimed at identifying and synthesizing the costs of COVID-19 on sociocultural issues in Bangladesh. For this purpose, we conducted a systematic search in MEDLINE, PubMed, ProQuest, Web of Science, Scopus and Google Scholar up to August 2021. Studies related to the costs of COVID-19 were identified, tabulated, analyzed, and synthesized by using a thematic approach. Our final synthesis of 19 studies resulted in five analytical themes: (i) disruption in education, (ii) loss of everyday social interaction, (iii) increase of “new poor” and suicide, (iv) rise of violence against women, and (v) worsening the life of refugees. Our findings showed that the costs of disruption in education, loss of everyday social interaction, and increase of “new poor” and suicide were more evident. Finally, we recommend the government and the community to adopt some integrated actions and policies to combat the problems in improving Bangladeshi sociocultural situations.

## Introduction

In December 2019, a novel coronavirus emerged in Wuhan, China (COVID-19), and rapidly spread across the globe to several other countries, becoming a threat to public health globally (McFadden et al., 2020). Within weeks, the World Health Organization designated the virus a global pandemic, and all countries were urged to take “urgent and aggressive action” to reduce the risk of viral contamination and fatalities (World Health Organization, 2021). Globally, the severe acute respiratory syndrome coronavirus 2 (SARS-CoV-2) is responsible for more than 226 million confirmed cases and nearly 4.6 million deaths in September of 2021 (Worldometer, 2021).

Bangladesh, a South Asian densely populated developing country with a total population of nearly 166 million estimated in 2021 (World Population Review, 2021) is facing untold challenges due to the outbreak of COVID-19. On March 8, 2020, the Institute of Epidemiology, Disease Control and Research (IEDCR), a research institute for monitoring COVID-19 in Bangladesh, first confirmed coronavirus cases in Bangladesh (Paul, 2020; TBS Report, 2020). The high density of population makes Bangladesh more vulnerable to the spread of the virus compared with other countries, where population density is lower (Islam, Ali et al., 2020).

As the pandemic evolves, the discussion on sociocultural consequences becomes more relevant. In line with the definition, the term sociocultural includes a combination of social and cultural factors (Merriam-Webster, n.d.). The issues related to social and cultural are used interchangeably and for this reason, these two terms are mixed as sociocultural. Sociocultural factors refer to those aspects of the social environment which are a direct result of the intersection between the cultural underpinnings of society (such as a collective system of values, beliefs, and thoughts) and its social processes and organizational mechanisms (such as social interaction and relationships, and institutional dynamics) (Shier et al., 2011). Thus, sociocultural factors generally include poverty, inequality, gender, religion, buying habits, education level, family size and structure, and population density (APA Dictionary of Psychology, n.d.; Globalization Partners International, n.d.; LaPoint et al., 2010).

The COVID-19 pandemic has had far-reaching consequences beyond the spread of the disease itself and efforts to quarantine it, including cultural and social implications (Wikipedia, 2021). A social crisis is considered much more than a health crisis, and the pandemic is attacking societies at their core (UN DESA, 2021). The COVID-19 lockdown has caused immense miseries and sorrows in everyone’s life, especially students’ education has been disrupted, and 3.7 million people have emerged as the “new poor” (Barkat et al., 2020).

Considering the loss and crisis of COVID-19 pandemic discussed above from a sociocultural perspective, a systematic review would benefit to assess and evaluate the current as well as tackling future pandemic consequences from both cross-country and cross-cultural perspectives. Therefore, to move forward with a systematic review, Bangladesh would be an impactful choice as it belongs to the list of developing countries, which also has suffered subsequently from the COVID-19 pandemic. However, a few literature reviews were undertaken in terms of COVID-19 in Bangladesh, and these were limited to identify the physical transmission rate, economic loss, food shortage and mental stress. For instance, most studies revealed the infection rate of coronavirus, human-to-human transmission, the number of new cases and mortalities (Islam, Islam et al., 2020; Rahman et al., 2021; Shahera et al., 2020). Two studies focused on the loss of economy (Mohiuddin, 2020) and tourism (Deb & Nafi, 2020) due to the pandemic. The reduction of food supply was found more evident due to labor shortages (Zabir et al., 2021). In addition, a study, which highlighted mental stress, included mild to severe symptoms of depression, anxiety and stress, and these were related to socioeconomic, behavioral and health factors (Al Mamun et al., 2021). To the authors’ best of knowledge, no attempt has been made to review sociocultural costs or the impact of COVID-19 in Bangladesh so far. Therefore, considering the importance of sociocultural issues and the paucity of information, this review paper aims to identify and synthesize the sociocultural costs of COVID-19 in Bangladesh. The synthesized review on the sociocultural costs of COVID-19 in Bangladesh would contribute to a wider group of readers and policymakers in academia both from a cross-country and cross-cultural perspectives. To achieve this aim, we synthesized studies resulted from a structured literature search.

## Methods

This review follows the recently updated PRISMA guidelines for reporting systematic reviews (Page et al., 2021) as this guideline shows a step-by-step process to report the review.

### Search Strategies

This review used five electronic databases: MEDLINE, PubMed, Web of Science, Scopus, and ProQuest Sociological Abstract, along with additional searches incorporated Google Scholar and Google. The search was completed between April to August 2021 as the case of coronavirus in Bangladesh started in March 2020. Studies from additional searches were included as studies identified from databases were not enough to find the real picture. The search included medical subject headings (MeSH) and text words for social, impact, and coronavirus. The Population, Intervention, Comparison and Outcome (PICO) model was used to develop search terms from the research question. The utilizing search strategy included keywords: “COVID-19,” “COVID-19 pandemic,” “coronavirus,” “sociocultural crisis,” “sociocultural impact” and “Bangladesh.” Detailed search strategies for each database are shown in Table 1.

**Table 1.** Search Terms and Items Found for Databases.

Search	Query	Items found
Medline		
#1	COVID-19.mp.	595,347
#2	Coronavirus* pandemic.mp.	7,813
#3	1 or 2	597,227
#4	Sociocultural impact.mp.	50
#5	Sociocultural crisis.mp.	1
#6	Sociocultural costs.mp.	2
#7	Education impact.mp.	1,164
#8	(Domestic or gender violence).mp.	377,073
#9	Religious impact.mp.	25
#10	Suicide.mp.	323,577
#11	4 or 5 or 6 or 7 or 8 or 9 or 10	695,637
#12	Bangladesh*.mp.	70,756
#13	3 and 11 and 12	147
PubMed		
#1	Coronavirus	246,142
#2	COVID-19	325,269
#3	COVID-19 pandemic	300,358
#4	(((Coronavirus) OR COVID-19)) OR COVID-19 pandemic	360,566
#5	Sociocultural[All Fields] AND “costs”[All Fields]	9,203
#6	Sociocultural[All Fields] AND “impact”[All Fields]	28,315
#7	Sociocultural impact	28,315
#8	Socioeconomic crisis	33,316
#9	Suicide	147,943
#10	Religious impact	62,901
#11	Domestic or gender violence	324,560
#12	Educational impact	341,665
#13	((suicide) AND ((((religious impact) OR (domestic or gender violence)) OR educational impact) OR ((((sociocultural[All Fields] AND “costs”[All Fields])) OR (sociocultural[All Fields] AND “impact”[All Fields])) OR sociocultural impact))) AND socioeconomic crisis	3,524
#14	Bangladesh	73,494
#15	((((((Coronavirus) OR COVID-19)) OR COVID-19 pandemic)) AND (((suicide) AND ((((religious impact) OR (domestic or gender violence)) OR educational impact) OR ((((sociocultural[All Fields] AND “costs”[All Fields])) OR (sociocultural[All Fields] AND “impact”[All Fields])) OR sociocultural impact))) AND socioeconomic crisis)) AND Bangladesh	190
Web of Science		
#1	(ALL=(COVID-19)) OR ALL=(Coronavirus* pandemic)	269,358
#2	((((((ALL=(sociocultural impact)) OR ALL=(sociocultural crisis)) OR ALL=(sociocultural costs)) OR ALL=(education impact)) OR ALL=(domestic or gender violence)) OR ALL=(religious impact)) OR ALL=(suicide)	836,595
#3	ALL=(Bangladesh*)	92,718
#4	#1 AND #2 AND #3	430
Scopus		
#1	“covid-19” OR “covid-19 pandemic” OR “coronavirus”	527,530
#2	“sociocultural impact” OR “sociocultural crisis” OR “sociocultural costs” OR “education impact” OR “domestic or gender violence” OR “religious impact” OR “suicide”	413,365
#3	“Bangladesh”	352,921
#4	#1 AND #2 AND #3	950
ProQuest Sociological Abstract		
S1	Coronavirus OR Covid-19 OR (COVID-19 pandemic)	8,611
S2	Suicide OR (religious impact) OR (Gender, Violence AND the Social Order) OR (educational impact) OR (sociocultural costs) OR (sociocultural impact) OR (sociocultural crisis)	151,197
S3	Bangladesh	10,432
S4	S1 AND S2 AND S3	273

**Figure 1.**
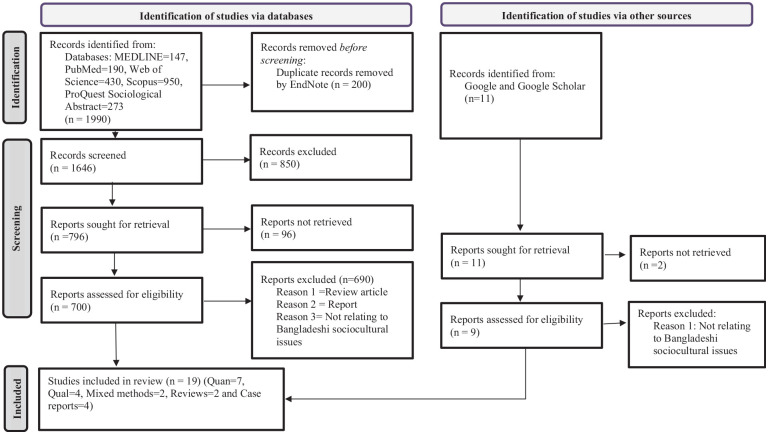
PRISMA flowchart for searches of databases and records.

The study adopted the PRISMA flowchart (Figure 1), which has recently been updated by Page et al. (2021) to identify the included studies systematically for this review. The search process retrieved 2,001 items of literature (Figure 1). Subsequently, the Endnote reference management software (version X9) and manual verification process were carried out to verify and eliminate 200 duplicate studies. From the search results, all potential articles for full-text review were screened using the titles and abstracts (*N* = 1646). To study relevant studies from databases, the title of each study was first read and then the abstract. If the abstracts were found relevant to the sociocultural costs of COVID-19, we read the whole study and included them in the review. Studies were read by the four authors (SKC, MRK, TR, and MHH) for crosschecking of information.

### Eligibility Criteria

Inclusion and exclusion criteria are listed in Table 2. The study used peer-reviewed studies including original articles, reviews and case reports, which focused on social and cultural issues in Bangladesh. In addition, studies, which were published in the English language, were included as these studies were accessible to international readers. However, we did not consider posters, conference abstracts, supplements, newspaper articles and reports to be included in the review as these are not peer-reviewed.

**Table 2.** Inclusion and Exclusion Criteria.

Factor	Inclusion criteria	Exclusion criteria
Purpose	Focus on the sociocultural impact of coronavirus, such as disruption of education, poverty, lack of social interaction and violence	Focus on the non-sociocultural impact of coronavirus, such as physical transmission, economic instability and food scarcity
Text	Full-text articles	Posters, conference abstracts, supplements, newspaper articles, government and NGO reports
Format	English language, peer-reviewed and no time-limit	Bangla language and non-peer-reviewed
Country	Bangladesh	Non-Bangladesh

### Assessing the Quality of the Included Literature

The quality of the selected studies was assessed by using different assessment criteria as this review included studies from different approaches. The quality of the qualitative studies was assessed by using the critical appraisal skill program (CASP) checklists (CASP, 2018), which assess the rigor, credibility and relevance of the qualitative study. The CASP contains 10 items, with items 1 to 9 were questions with possible answers of “Yes,” “No,” or “Can’t tell” (Table 3). Item 10 required discussion among assessors.

**Table 3.** Quality Appraisal of the Qualitative Studies.

Author(s), year, country	1. Was there a clear statement of the aims of the research?	2. Is qualitative Methodology appropriate?	3. Was the research design appropriate to address the aims of the research?	4. Was the recruitment strategy appropriate to the aims of the research?	5. Was the data collected in a way that addressed the research issue?	6. Has the relationship between researcher and participants been adequately considered?	7. Have ethical issues been taken into consideration?	8. Was the data analysis sufficiently rigorous?	9. Is there a clear statement of findings?	10. How valuable is the research?	Total score (%) and quality
Ahmed et al. (2020), Bangladesh, Kenia, Nigeria and Pakistan	Y	Y	Y	Y	Y	Y	Y	Y	Y	The research is highly valuable as it shows the impact of COVID-19 on various social issues of slum dwellers and implies the policy implications.	9/9 (100.0) High
Dutta and Smita (2020), Bangladesh	Y	Y	Y	Y	Y	Y	N	Y	Y	The research is highly valuable as it shows the educational impact of COVID-19 and addresses the educational policy implications.	8/9 (88.9) High
Ela et al. (2021), Bangladesh	Y	Y	Y	Y	Y	Y	Y	Y	Y	The research is highly valuable as it shows the impact of COVID-19 on the academic life and career of students.	9/9 (100.0) High
Guglielmi et al. (2020), Bangladesh (Qualitative part)	Y	Y	Y	Y	Y	Y	N	Y	Y	The research is valuable because it focuses on the experiences of displaced Rohingya adolescents during COVID-19.	8/9 (88.9) High (Average score and quality: 8 (80.8) High)
Islam, Islam et al. (2020), Bangladesh (Qualitative part)	Y	Y	Y	Y	Y	Y	N	Y	Y	The research is highly valuable as it focuses on several healthcare access barriers during COVID-19.	8/9 (88.9) High (Average score and quality: 6.5 (65.0) Good)
Uddin (2021), Bangladesh	Y	Y	Y	Y	Y	Y	N	Y	Y	The study is very valuable because it emphasizes women’s work-life balance during COVID-19.	8/9 (88.9) High

The cross-sectional quantitative studies were assessed by using the Critical Appraisal Checklist (CEBM, 2014), which includes 12 questions with possible answers of “Yes,” “No,” or “Can’t tell” to assess the research design, selection of the subjects and representatives, the reliability of the measurement and the statistical analysis (Table 4). The qualitative part of the mixed methods studies was assessed with the CASP checklist (Table 3) and the quantitative part with the Critical Appraisal Checklist (Table 4). The review papers were assessed with the CASP checklist (Table 5). Finally, the critical appraisal developed by Roever and Ocke Reis (2015) was used to assess the case reports (Table 6).

**Table 4.** Quality Appraisal of the Quantitative Cross-sectional Studies.

Author (s), year, country	1. Did the study address a clearly focused issue?	2. Is the research method (study design) appropriate for answering the research question?	3. Is the method of selection of the subjects (employees, teams, divisions, organizations) clearly described?	4. Could the way the sample was obtained introduce (selection) bias?	5. Was the sample of subjects representative with regard to the population to which the findings will be referred?	6. Was the sample size based on pre-study considerations of statistical power?	7. Was a satisfactory response rate achieved?	8. Are the measurements (questionnaires) likely to be valid and reliable?	9. Was the statistical significance assessed?	10. Are confidence intervals given for the main results?	11. Could there be confounding factors that haven’t been accounted for?	12. Can the results be applied to your organization?	Total score and quality
Bodrud-Doza et al. (2020), Bangladesh	Y	Y	Y	No bias	Y	Y	Y	Y	Y	Y	Y	Y	11/11 (100.0) High
Emon et al. (2020), Bangladesh	Y	Y	N	It introduces bias as non-probability sampling method was undertaken to select participants.	Y	N	C	Y	N	N	N	Y	5/11 (45.5) Poor
Guglielmi et al. (2020), Bangladesh (Quantitative part)	Y	Y	Y	No bias	Y	Y	Y	C	Y	N	C	Y	8/11 (72.7) Good
Hamadani et al. (2020), Bangladesh	Y	Y	Y	No bias	Y	Y	Y	Y	Y	Y	Y	Y	11/11 (100.0) High
Islam, Islam et al. (2020), Bangladesh (Quantitative part)	Y	Y	Y	No bias	C (no information was found)	N	C (no information was found)	Y	N	N	N	Y	5/11 (45.5) Poor
Kamrujjaman et al. (2021), Bangladesh	Y	Y	Y	No bias	Y	Y	C	Y	Y	N	Y	Y	9/11 (81.8) High
Rayhan and Akter (2021), Bangladesh	Y	Y	Y	No bias	Y	N	Y	Y	Y	Y	Y	Y	11/11 (100.0) High
Shammi et al. (2020), Bangladesh	Y	Y	Y	No bias	Y	C (no information was found)	Y	Y	Y	Y	Y	Y	10/11 (90.9) High
Truelove et al. (2020), Bangladesh	Y	Y	Y	No bias	Y	Y	C	Y	Y	Y	C	Y	9/11 (81.8) High

**Table 5.** Quality Appraisal of the Review Studies.

Author(s), year, country	1. Did the review address a clearly focused question?	2. Did the authors look for the right type of papers?	3. Do you think all the important, relevant studies were included?	4. Did the review’s authors do enough to assess quality of the included studies?	5. If the results of the review have been combined, was it reasonable to do so?	6. What are the overall results of the review?	7. How precise are the results?	8. Can the results be applied to the local population?	9. Were all important outcomes considered?	10. Are the benefits worth the harms and costs?	Total score (%) and quality
Begum et al. (2020), Bangladesh	Y	Y	N	N	Y	A substantial impact of COVID-19 was identified on different socioeconomic sectors, such as business, pharmaceuticals, education, etc.	This paper shows a narrative description about the social, economic and health impact of COVID-19.	Y	Y	C There is insufficient information here to say either way. Harms and costs were not measured.	5/8 (62.5) Good
Mamun (2021), Bangladesh	Y	Y	Y	N	Y	Assessed the prevalence and risk factors of suicidal behavior	Suicidal behavior was related to sociodemographic, behavior and health-related, COVID-19 pandemic-related, and psychopathological issues	Y	Y	C There is insufficient information here to say either way. Harms and costs were not measured.	6/8 (75.0) Good

**Table 6.** Quality Appraisal of the Case Reports.

Author(s), year, country	1. Did the study address a clearly focused question/issue?	2. Is the study design appropriate for answering the research question?	3. Was the study well-defined protocol?	4. Are both the setting and the subject’s representative with regard to the population to which the findings will be referred?	5. Is the researcher’s perspective clearly described and taken into account?	6. Are the methods for collecting data clearly described?	7. Are the methods for analyzing the data likely to be valid and reliable? Are quality control measures used?	8. Was the analysis repeated by more than one researcher to ensure reliability?	9. Are the results credible, and if so, are they relevant for practice? Are results easy to understand?	10. It was clinically relevant outcomes?	11. Are the conclusions drawn justified by the results?	12. Are the findings of the study transferable to other settings?	Total score (%) and quality
Banik et al. (2020), Bangladesh	Y	Y	Y	Y	Y	Y	N	Y	Y	C (no indication was found about this)	Y	Y	10/12 (83.3) High
Bhuiyan et al. (2021), Bangladesh	Y	Y	Y	Y	Y	Y	N	Y	Y	C (no indication was found about this)	Y	Y	10/12 (83.3) High
Mamun et al. (2020), Bangladesh	Y	Y	Y	Y	C (no indication about this was found)	N (methodology section is not included)	N	Y	Y	C (no indication was found about this)	Y	Y	8/12 (67.0) Good
Mamun and Griffiths (2020), Bangladesh	Y	C (no indication about this was found)	N	Y	Y	N (methodology section is not included)	N	Y	Y	C (no indication was found about this)	Y	Y	7/12 (58.3) Poor

To count a total score and quality of the papers (shown in the right column of Tables 3–6) included in this systematic review, the following procedures were followed for all appraisals: (a) the total score of each table was calculated, and the achieved score was divided by the total score, and then the score was converted into a percentage to categorize the quality of the papers; and (b) the quality of the papers was categorized into three based on the percentage distribution and range: (i) 80% to 100% = high, 60% to 79% = good, and <60% = poor. Qualitative answers were not counted for scoring, and explanatory hints were provided for each question. In addition, the total score and quality for mixed methods papers were made after combining scores from both qualitative and quantitative parts, which were presented as average score and quality in Table 3.

### Data Extraction

Study characteristics and key findings were extracted and tabulated according to the guidelines of Ma et al. (2017). Major characteristics included (1) author(s), year of publication, and the country where the study was conducted, (2) aims of the study, (3) study design, (4) sampling method and participants, (5) focus and significance findings, and (6) quality. To begin with, the studies were separately grouped and summarized based on qualitative or quantitative design. Likewise, data obtained from the qualitative or quantitative components of the mixed methods studies were included in the relevant group. The data extraction tables for studies subject to double data extraction were reviewed by a third reviewer (TR), then discussed with the primary reviewers (SKC and MRK). The level of agreement for data extraction was found to be good. The characteristics and key findings of these studies were summarized and categorized in Table 7.

**Table 7.** Study Characteristics and Summary of Findings From the Included Studies.

Author, year, country	Aims	Study design	Sampling method and participants	Focus and significant findings
Ahmed et al. (2020), Bangladesh, Kenya, Nigeria, and Pakistan	To understand and respond to the health needs of slum communities	Qualitative	Purposive sampling, and patient medicine vendors, polio workers, clinical officers, nurses, community health workers, pregnant women and women with children	• Interrupted access to basic needs • Reduced availability of basic services • Price hike of basic needs • Increased patronage of locally available services • Increased gender-based violence
Banik et al. (2020), Bangladesh	To find out the major concerns of Rohingya refugees during the pandemic	Commentary	Secondary sources	• Lack of access to services for life-saving, such as food, drinkable water, shelter and health services • Increase of rumor and violence against women
Begum et al. (2020), Bangladesh	To deliver a comprehensive overview of the observed and possible impacts that could appear in the coming days	Narrative review	The electronic search strategy was used to explore studies and narratively review them	• A substantial impact of COVID-19 was identified on different socio-economic sectors in Bangladesh, such as local businesses and farms, pharmaceuticals, educational system, and banking.
Bhuiyan et al. (2021), Bangladesh	To narrate some cases of suicide due to COVID-19 lockdown and economic factors in Bangladesh	Letter to the editor	Eight cases of COVID-19 related suicide were published in national dailies	• Denial of financial support from the local government authorities • Food insecurity • Increase of unemployment • Rebuke by parents
Bodrud-Doza et al. (2020), Bangladesh	To understand the probable psychological, socioeconomic and environmental impact of the COVID-19 outbreak in Bangladesh	Quantitative	Simple random sampling, and participants belonged to different social categories	• Poor governance in healthcare • Lack of healthcare treatment • Fear and anxiety and mental stress • Migration • Hindering formal education • Social conflict • Price hike of essentials • Loss of livelihood • Food insecurity
Dutta and Smita (2020), Bangladesh	To explore the impact of the COVID-19 pandemic on tertiary education in Bangladesh	Qualitative	Convenience sampling, and university students	• Disruption in students’ learning • Decrease in motivation and study hours • Disruption of social networking and ties • Social stress • Laws of social interaction • Barriers to social skill development
Ela et al. (2021), Bangladesh	Focuses on displaced Rohingya adolescents’ experiences during COVID-19	Qualitative	Purposive sampling, and university teachers and students	• An extended closure is responsible for the delayed graduation • Mounting mental stress and frustration among students • Unequivocally opposed the online education platform due to scarcity of the resources • Unequal access and opportunities
Emon et al. (2020), Bangladesh	To identify the effects of the COVID-19 pandemic on the education system of Bangladesh and its possible solutions	Quantitative	Online survey, and students	• Disruption of education • Out school learning deprivation
Guglielmi et al. (2020), Bangladesh	To explore vulnerabilities faced by Rohingya adolescents living in Cox’s Bazar during the COVID-19 pandemic	Mixed methods	Random sampling, and Rohingya adult adolescents and older adolescents	• Rohingya adolescents’ health status, food insecurity, educational, economic marginalization and bodily integrity risks
Hamadani et al. (2020), Bangladesh	To determine the immediate impact of COVID-19 lockdown orders on women and their families in rural Bangladesh	Quantitative	Random sampling and women	• Reduction in paid work • Experience of food insecurity • Physical violence and Humiliation
Islam, Islam et al. (2020), Bangladesh	To analyze healthcare, social and economic challenges	Mixed methods	Secondary sources, and observation, and people from all levels	• Lack of awareness • Improper knowledge, attitude to and practice of rules • Poverty and precarious employment • Housing problem • Crowded transport during holidays and festivals • Barriers to religious ceremonies due to social distancing • Gender-based discrimination • Domestic violence
Kamrujjaman et al. (2021), Bangladesh	To forecast the COVID-19 obligation among the Rohingya refugees of Bangladesh	Quantitative	Modeling, and Rohingya refugee	• Reasonable control over the transmission of the COVID-19 disease among Rohingya
Mamun and Griffiths (2020), Bangladesh	To identify the possible strategies to prevent suicide during COVID-19	Case report	Not reported	• Mental healthcare is also needed for patients confirmed as having COVID-19 • Avoid misinformation and online-based mental health intervention programs as a way of promoting more reliable information
Mamun et al. (2020), Bangladesh	To report a novel reason for suicide and seemingly COVID-19 related negligence in getting treatment by Bangladeshi healthcare providers	Case report	Not reported	• Committed suicide by a woman as she didn’t get treatment by the hospital healthcare staff due to the fear of COVID-19 transmission
Mamun (2021), Bangladesh	To review the Bangladeshi COVID-19 related suicide studies for the first time	Review	The PRISMA guideline was followed; five databases, such as PubMed, Scopus, PsycINFO, Web of Science, CINAH were searched; and nine types of literature were included	Factors concerning suicidal behavior included: • Socio-demographic variables • Behavior and health-related variables • COVID-19 pandemic-related variables • Psychopathological variables
Rayhan and Akter (2021), Bangladesh	To explore the prevalence and associated factors of IPV (Intimate Partner Violence) amid the COVID-19 pandemic	Quantitative	Survey, and women (16–49)	• The prevalence of IPV was 45.29% • Associated factors of IPV were types of marriage, area of residence, employment status, age and education
Shammi et al. (2020), Bangladesh	To understand the public perception of socio-economic crisis and human stress in resource-limited settings of Bangladesh during the COVID-19 outbreak	Quantitative	Online survey, and Bangladeshi participants having age above 18,	• Increase of migration from urban to rural • Increase of unemployment • Decrease of food supply • Increase of social conflict • Vulnerability of children • Disruption of the educational system • Increase of family conflicts
Truelove et al. (2020), Bangladesh	To explore the potential impact of severe acute respiratory syndrome coronavirus 2 (SARS-CoV-2) in the Kutupalong-Balukhali Expansion Site	Quantitative	Stochastic Susceptible Exposed Infectious Recovered (SEIR) mathematical model, and Rohingya refugee	• Possibility of the outbreak of COVID-19 within the expansion site with Rohingya • A large-scale outbreak is very likely in this setting after a single infectious • The need for hospitalization may exceed the existing capacity
Uddin (2021), Bangladesh	To address work-family issues of working women in the distinct Bangladeshi socio-cultural context	Qualitative	Snowball sampling, and women	• Lack of available time, sociocultural and family norms, and gender stereotypes are major challenges for Bangladeshi women in managing work-life balance during COVID-19

### Data Analysis and Synthesis

This study included results from both qualitative and quantitative sides. Meta-analysis of the results of quantitative studies was inappropriate due to the heterogeneous nature of the studies, such as randomized control trial (RCT) and cross-sectional. So, thematic synthesis was used to synthesize the secondary results. There are trends of using thematic synthesis for social research (Ryan et al., 2018). Thematic synthesis is considered as an adaptation to secondary data synthesis of “thematic analysis” and offers a range of established methods and techniques for the identification and development of analytic themes in first-hand data (Thomas & Harden, 2008). As the included studies of this study used approaches in qualitative and quantitative, data were identified, tabulated, analyzed, and synthesized using a thematic approach and presented as narratives.

This synthesis approach has three stages of data analyses: Free line-by-line coding, organization of “free codes” to construct “descriptive” themes and the development of “analytical” themes. Two reviewers (SKC and MRK) independently coded each line of verbatim text labeled “results” or “findings” within the 18 studies. The text included participant quotations, themes, subthemes and findings of the original authors. Afterward, discrepancies of codes were minimized with the presence of all authors, and five themes were generated from codes or subthemes (Table 8). Two reviewers (TR and MHH) crosschecked themes concerning the review question to support the robustness of the analytical approach. A computer-aided software called NVivo 12 (QSR International Pty Ltd., 2022) was used to manage data.

**Table 8.** Analytical Themes Based on Categories or Descriptive Themes.

Analytical themes	Categories/descriptive themes	References
Disruption in education	Long closure of the educational institution	Begum et al. (2020), Bodrud-Doza et al. (2020), Dutta and Smita (2020), Ela et al. (2021), Emon et al. (2020), Shammi et al. (2020)
	Remote study	
	Consequences of remote study	
Loss of everyday social interaction	Cancellation of prayers	Begum et al. (2020), Dutta and Smita (2020), Emon et al. (2020), Guglielmi et al. (2020), Hamadani et al. (2020), Truelove et al. (2020)
	Isolate treatment of children	
	Social stigma	
Increase of “new poor” and suicide	Increasing poverty	Ahmed et al. (2020), Begum et al. (2020), Bhuiyan et al. (2021), Bodrud-Doza et al. (2020), Emon et al. (2020), Hamadani et al. (2020), Islam, Islam et al. (2020), Mamun (2021), Mamun et al. (2020), Mamun and Griffiths (2020), Shammi et al. (2020)
	Prone to suicide	
	Increasing social conflicts	
Rise of violence against women	Domestic violence	Begum et al. (2020), Hamadani et al. (2020), Islam, Islam et al. (2020), Rayhan and Akter (2021), Shammi et al. (2020), Uddin (2021)
	Intimate partner violence	
	Gender-based violence	
	Youth vulnerability	
Worsening the life of refugees	Prediction about the infection rate	Banik et al. (2020), Guglielmi et al. (2020), Kamrujjaman et al. (2021), Truelove et al. (2020)
	Lack of everyday livings	
	Rumor about COVID-19	
	Gender differences in food consumption	
	Gender differences in getting married	
	Challenge of maintaining COVID restrictions	

## Results

### Study Characteristics

As shown in Figure 1, the review includes 19 studies out of 2001 following the inclusion criteria. Five categories of studies were found crucial to be included in the review: (i) quantitative (seven), (ii) qualitative (four), (iii) mixed methods (two), (iv) reviews (two), and (v) case reports (four). All studies included in the review focused on Bangladeshi sociocultural issues, and these were published between 2020 and 2021.

### Quality Assessment

Following the assessment of 19 studies, 12 were found high, five were good, and two were poor in quality (Tables 3–6). Studies with good and poor qualities lacked proper methodological justification and analysis process. The quality of the studies included for the review ranged from 5 to 12 out of a possible 12 scores (mean = 8.7).

### Description of Themes

Five key themes related to the sociocultural costs of coronavirus emerged from the analysis of studies included in this review. The themes are (i) disruption in education, (ii) loss of everyday social interaction, (iii) increase of “new poor” and suicide, (iv) rise of violence against women, and (v) worsening the life of refugees.

### Theme 1: Disruption in Education

This theme included six studies (Begum et al., 2020; Bodrud-Doza et al., 2020; Dutta & Smita, 2020; Ela et al., 2021; Emon et al., 2020; Shammi et al., 2020), four of which were good qualities, which examined the impact of educational institutions closure on Bangladeshi students.

In the mid of March 2020, the government of Bangladesh declared the termination of schools, colleges and universities until further notice comes for reopening (Begum et al., 2020; Dutta & Smita, 2020; Ela et al., 2021). Although some private schools and universities (23%) were continuing with online learning, the vast section of educational institutions, such as government schools, colleges or universities remained closed due to not having uninterrupted internet facilities, which are identified in a quantitative study conducted by Emon et al. (2020). That is why students continued their studies at homes. However, a qualitative study conducted by Dutta and Smita (2020) emphasized that pupils were being demotivated to continue their studies from home as they did not have any specific guidelines to follow. Some university students were accustomed to studying at the library in a quiet situation instead of home, but they faced disruption of home study. An excerpt identified by Dutta and Smita (2020, p. 57) showed that “My house is noisy. So, I could never study at home. I used to study at the library or seminars in my department. I have been home for a long time now, but I cannot read.”

In addition, students, on the one hand, could not meet with teachers and peer mates to discuss such academic matters (Begum et al., 2020; Bodrud-Doza et al., 2020; Dutta & Smita, 2020), on the other hand, they were deprived of participating physical and social activities (Bodrud-Doza et al., 2020).

Thus, the consequences of on-spot study closure are divided into two: academic and non-academic issues. In the first case, two studies including one review and one quantitative study identified that all final examinations of students were postponed and consequently delayed from timely completion of studies and traveling abroad for higher studies (Begum et al., 2020; Emon et al., 2020). The non-academic influences include being less likely to get a job at the right time (Begum et al., 2020). Furthermore, students now cannot talk freely, join in a group study and make fun with friends due to the loss of social interaction (see Theme 2 for details) that triggered them to spend more time on social sites, such as using Facebook, watching television or YouTube and playing games on computer or mobile (Dutta & Smita, 2020; Emon et al., 2020). In addition, the younger generation was getting involved in social crimes, such as begging, child labor, and sex work (Emon et al., 2020), and primary school children, in a quantitative study, were found at extreme risk of abuse if their parents were quarantined (Shammi et al., 2020).

Therefore, long delays in studies not only delay study completion, but also lessen the job entry probabilities, daily social interactions and the normal student life of the youth.

### Theme 2: Loss of Everyday Social Interaction

This theme consisted of six reviewed studies (Begum et al., 2020; Dutta & Smita, 2020; Emon et al., 2020; Guglielmi et al., 2020; Hamadani et al., 2020; Truelove et al., 2020), of which four were high in quality. The reviewed studies under this current theme identified that the loss of everyday social interaction during COVID-19 in Bangladesh was becoming a predominant fact. The loss of social interaction was noticed among all classes of people, which made them disrupted in many situations.

A qualitative study from Dutta and Smita (2020) and two quantitative studies from Truelove et al. (2020) and Hamadani et al. (2020) found that the outbreak of COVID-19 has brought difficulties in the social interaction process. Dutta and Smita (2020) conducted their study on university students and found that students were under pressure to miss campus interactions with teachers and friends for face-to-face academic and non-academic affairs. Another quantitative study undertaken with students by Emon et al. (2020) found similar evidence to Dutta and Smita (2020). Both studies reported that the lack of interaction as a result of isolation and communication barriers caused emotional distress to most students.

A narrative review from Begum et al. (2020) showed that the common people (e.g., patients and their family members, health workers and community people at large) were also unable to continue their social interaction habits and practices like everyday time. Due to cultural mobility restrictions, people appeared to be the most affected by lockdown orders, lamenting a loosening of friendships (Guglielmi et al., 2020).

Begum et al. (2020) found that in many cases COVID-19 infected people were treated at COVID-19 isolation centers or hospitals instead of home; and, due to safety precautions, family members had to leave the infected person at an isolation center or hospital. A quantitative study from Shammi et al. (2020) had shown that staying in isolation centers for weeks or more creates further loneliness and anxiety for both the infected as well as for their family members. This isolation and social distance from the infected person led to the loss of social contact with close people, such as family members, relatives, and friends (Emon et al., 2020). Begum et al. (2020) argued that not only patients and their family members but also healthcare workers lost daily social interaction. The healthcare workers who treated patients and became infected were criticized and stigmatized by the community people (Begum et al., 2020).

Begum et al. (2020) further showed that the pandemic restrictions minimized religious activities, such as cancellation of the prayers in mosques, temples and churches, which also impacted the loss of everyday social interaction process.

Thus, it is clear from the findings that social isolation was evident among students, mass people, people with illness, healthcare staff and religious devotees.

### Theme 3: Increase of “New Poor” and Suicide

This theme included 11 studies (Ahmed et al., 2020; Begum et al., 2020; Bhuiyan et al., 2021; Bodrud-Doza et al., 2020; Emon et al., 2020; Hamadani et al., 2020; Islam, Islam et al., 2020; Mamun, 2021; Mamun et al., 2020; Mamun & Griffiths, 2020; Shammi et al., 2020), which reported the increase of “new poor” and suicide as a result of the long-term COVID-19 outbreak in Bangladesh. The new category of “new poor” refers to the Bangladeshi vulnerable non-poor who have fallen below the poverty line due to the impact of the COVID-19 crisis, but they were not poor before the pandemic (Rahman et al., 2020).

Several studies (Bhuiyan et al., 2021; Hamadani et al., 2020; Islam, Islam et al., 2020; Shammi et al., 2020) depicted that the introduction of COVID-19 pandemic increases the poverty situation among the people of Bangladesh. Quantitative studies from Hamadani et al. (2020) and Shammi et al. (2020) characterized that the poverty situation is increasing due to displacement from jobs, reduction in paid hours and an increase in the unemployment rate. Another quantitative study (conducted on students) from Emon et al. (2020) showed that the increasing crisis in the job sector had significantly affected the fresh graduates, who wanted to involve in income-generating activities after their graduation. Hence, the job crisis is not only narrowing the income-generating opportunities of the workforce at present but also preventing potential manpower (fresh graduates) from joining the productive sectors (Emon et al., 2020), which indicates the creation of new poor.

A quantitative study from Bodrud-Doza et al. (2020) reported that the unemployment situation had also created tension, fear and anxiety among the affected people. A qualitative study conducted by Bhuiyan et al. (2021) found that eight people aged between 10 and 35 committed suicide due to economic hardship during the pandemic. Apart from economic hardship, fear of the COVID-19 transmission, social stigma, isolation, stress and loneliness also pushed people to commit suicide (Mamun, 2021; Mamun & Griffiths, 2020). A case study highlighted this issue and presented the evidence that

… COVID-19-related negligence in getting treatment by Bangladeshi healthcare providers. … a woman committed suicide at a hospital because she was not treated, and doctors and nurses suspected she was infected with COVID-19 and did not want to get infected themselves. (Mamun et al., 2020, p. 1)

Hence, the findings indicate that COVID-19 has increased the number of new poor and suicide rates in Bangladesh and that most victims are young people.

### Theme 4: Rise of Violence Against Women

This theme included six studies (Begum et al., 2020; Hamadani et al., 2020; Islam, Islam et al., 2020; Rayhan & Akter, 2021; Shammi et al., 2020; Uddin, 2021), of which five were high in quality, and examined the violence against women during COVID-19 in Bangladesh. The review process under the current theme revealed three types of violence, such as intimate partner violence (IPV), gender-based violence and domestic violence in Bangladesh during the COVID-19 pandemic. Studies included in this section indicated that these types of violence increased alarmingly as a form of discrimination against women during COVID-19.

A quantitative study conducted by Shammi et al. (2020) showed that subsequent and prolonged lockdown in Bangladesh during the COVID-19 intensified conflict among family members, especially between men and women resulting in IPVs. They found that the intensified conflict resulted in the physical and emotional assault against women (Shammi et al., 2020). A mixed methods study conducted by Islam, Islam et al. (2020), and a narrative review conducted by Begum et al. (2020) also found similar evidence of family conflict. Almost 45.29% of women experienced violence from their partners during the pandemic, and among them, 15.29% faced physical torture and 44.12% faced mental health-related torture (Rayhan & Akter, 2021). Using a random sampling method on women, a quantitative study conducted by Hamadani et al. (2020) also reported the same as Rayhan and Akter (2021). A study reported that of women who experienced gender-based violence, 42.2% of them faced mental health-related torture, 6.5% experienced physical torture and 3.0% experienced sexual torture by their partners (Hamadani et al., 2020).

Moreover, women faced gender-based discrimination during the pandemic, for example, the pressure of extra efforts required by women to manage increased household chores (Shammi et al., 2020). The economic hardship during the lockdown affected the mental well-being of earning members, and this also led them to conduct gender-based violence. Islam, Islam et al. (2020) further argued that working women, whose work was shifted to the home due to the lockdown, had experienced more gender-based discrimination and violence, for example, women needed to spend more hours doing household chores in addition to their regular jobs. Thus, maintaining a work-life balance was a challenge for women during the pandemic because they faced non-cooperation from their spouses to manage household activities before working for the industry, which affect their well-being (Uddin, 2021). Furthermore, due to staying at home during COVID-19 an upward trend of domestic violence among all social classes in Bangladesh was found by Begum et al. (2020), Islam, Islam et al. (2020). Women faced domestic violence in the form of verbal and physical abuse by the closest one living in the same house and sharing the same meal (Begum et al., 2020).

Apart from the violence against women, another alarming fact that has been revealed by Begum et al. (2020) has shown the increasing violence against young girls. The violence against the young girls was mostly reported by rape cases, which also included sexual abuse, physical torture and post-rape mental health issues of the victims. For instance, between January to June 2020, the number of rape incidence increased from 98 to 174 (Begum et al., 2020).

In short, there has been an increase in violence against women and girls by men in verbal and physical forms during the pandemic.

### Theme 5: Worsening the Life of Refugees

The theme of worsening the life of refugees incorporated four studies (Banik et al., 2020; Guglielmi et al., 2020; Kamrujjaman et al., 2021; Truelove et al., 2020), two of which were quantitative, one mixed methods, and one commentary studies.

The COVID-19 pandemic resulted in panic and significant health concern for people from minorities such as the refugees. The pandemic period was found to affect the high population density of refugee settlements with various profound consequences. A quantitative study has predicted that 92% of people could become infected after a single introduction of the virus (Truelove et al., 2020). In this concern, Rohingya refugees in Bangladesh were in the most vulnerable situation during the pandemic as they had a lack of access to food, drinkable water, shelter and health services (Banik et al., 2020; Kamrujjaman et al., 2021). In addition, lack of education, knowledge and social interaction among the Rohingya refugees was turning an already crisis into a major human disaster (Guglielmi et al., 2020). Due to their lack of education and health knowledge, they were also facing and trusting rumors about COVID-19, and violence against women and girls had been found to increase in number (Banik et al., 2020; Guglielmi et al., 2020). For example, there were widespread rumors among Rohingya refugees that anyone with coronavirus symptoms would be arrested, and as a result, many Rohingya Muslims took part in prayers in the belief that the virus would stop spreading (Banik et al., 2020).

A mixed methods study undertaken by Guglielmi et al. (2020) explored the gender differences in food consumption and marriage among adolescent Rohingyas during the COVID-19 period. For example, boys (16%) were less likely to starve than girls (23%) and at risk of early marriage (11%) compared to girls (19%) (Guglielmi et al., 2020). Moreover, previous research has shown that cultural norms and practices would also impact the Rohingya people to combat COVID-19. For example, isolating people, especially the elderly Rohingya, will be culturally challenging because the people in this community are accustomed to living together and sharing feelings for ages (Kamrujjaman et al., 2021). In addition, most of the current interventions, such as social distancing, contact tracing and isolation, good hygiene and better treatment in Intensive Care Units (ICUs) for critical cases will be challenging to implement in the camps (Truelove et al., 2020).

## Discussion

This systematic review aimed to identify and synthesize costs of COVID-19 on the sociocultural issues of Bangladesh. In the current systematic review of 19 studies, we found that the COVID-19 pandemic had several sociocultural costs, such as educational discontinuity, loss of social interaction, increase of poverty and suicide, rise of violence against women and worsening the life of refugee. These sociocultural costs resulted in the precarious lives of youth due to educational disruptions and the individualistic lives of the masses due to the loss of social ties. In addition, the lives of poor people, women and refugees became vulnerable and unequal due to the increase of poverty and violence, and the deterioration of living standards. The present study is the first systematic review and thematic synthesis in which the sociocultural costs of COVID-19 in Bangladesh has been contextualized from the existing literature, which is expected to be helpful for the social policy and practice.

The first key finding of this paper showed that due to COVID-19, the prolonged shutdown of educational institutions in Bangladesh and the online education system caused immense disruptions to students’ learning process, such as lack of concentration during distance learning and access to library resources. This finding appears similar to Debbarma and Durai (2021) who addressed that in India online learning creates concentration breaks for students during the pandemic. Owusu-Fordjour et al. (2020) found that in Ghana due to isolation, students did not have access to library resources and learning materials from home. The teaching and learning environment were also found disrupted by unanticipated noise disruption from the home environment both for students and teachers (Owusu-Fordjour et al., 2020). The findings anticipate that the academic future of students became uncertain in many ways. For example, the closure of educational institutions, delays in examinations and uncertainty of projecting future studies made the situation even worse in Bangladesh. Also, our review findings indicate that students who spent a long time on-screen during COVID-19 might harm the body and mind. The World Health Organization (2020) highlighted that excessive screen time replaces healthy behaviors and habits, such as physical activity and sleep routine and leads to potentially harmful effects, such as reduced sleep or day-night reversal, headaches, neck pain, myopia, digital eye syndrome and cardiovascular risk factors, including obesity, high blood pressure, and insulin resistance due to increase in sedentary time among adults. However, none of the studies included in this review highlighted how to resume the study amid the pandemic and beyond. Moreover, the online-based learning and isolation from social interaction involved students’ high involvement with social sites and electronic devices.

The second major finding of this study is that the COVID-19 pandemic caused a massive loss of social interactions not only for students but also for the community people. The same situation is also found in another study where it is reported that the usual ways in which individuals interact and obtain social support were severely disrupted (Long et al., 2022). Also, the findings of our review indicate that declaration on social distancing for a long time from the government’s side increased depression and anxiety among people which might make them more individualistic. The second finding corroborates with a recent study that shows that the impact of social isolation may be most hard felt for those who are usually socially active and more empathic (Sommerlad et al., 2021).

The third finding of our review showed that the new poor in Bangladesh during COVID-19 is increasing due to unemployment, low income-generating activities and fewer opportunities to involve new manpower in the production process (Emon et al., 2020; Hamadani et al., 2020). This new evolving poverty and its consequences were evidenced in other low-income countries as well. Findings from Sumner et al. (2020), further support the evidence of this current study and suggests that an economic depression due to COVID-19 may lead the world economy to 30 years back. Furthermore, this study also identified the social issue of committing suicide. Such findings also support some other scholarly works in India (Sripad et al., 2021) and Guyanese (Arora et al., 2020), where economic stress was found to be associated with a higher rate of suicides in this pandemic.

The fourth key finding of this study showed that the COVID-19 lockdown increased violence against women. Due to the prolonged lockdown, men had to stay at home for a long time, which in turn led them to engage in different abuse against women (Begum et al., 2020). Likewise, in India, a growing number of studies from Krishnakumar and Verma (2021), Maji et al. (2021), Nair and Banerjee (2021) have evidenced an increase in domestic violence, IPV and gender-based violence during the COVID-19 lockdown. Similarly, Baig et al. (2020), Haq et al. (2020) further showed that in Pakistan, women had to take more responsibility and stay longer hours with their abusive partners during this pandemic. The violence against women is worrisome during the COVID-19 lockdown that demands concerted efforts not only from the government and law enforcement agencies, but also growing awareness among mass people, especially among women to curve this shadow pandemic.

The final finding of this current study showed a worsening living situation of the Rohingya refugees in Bangladesh. The refugees generally have limited access to transportation, remote and poor healthcare, education, economic activities compared to the majority of the country’s population. The lockdown situation even made it worse for the refugees staying in Bangladesh such as introducing a gender difference in early marriage and food consumption (Guglielmi et al., 2020). This study finding showed the urgency of policy implications necessary to safeguard refugee adolescent trajectories in the context of COVID-19. The pandemic affects all segments of the population and is particularly detrimental to members of those social groups who are most at risk, including people living in poverty the elderly, people with disabilities, youth, and indigenous peoples (UN DESA, 2021).

### Strengths and Limitations

To contextualize the strength of this study, the results and discussion sections would contribute to global readers. Although the results section presents information only from Bangladeshi perspective, the discussion section compares and contrasts the results of the review with existing world literature. Therefore, to our consideration, countries from both developed and developing contexts may find this study significant while documenting the evidence and articulating prospective policies and planning to fight the sociocultural costs which arose from this current and may occur from any future pandemic. To minimize bias, we employed rigorous search methods, including an extensive and comprehensive search and data extraction by two independent reviewers. However, as only studies written in English were included, we may have missed studies published in Bengali, which would improve the insights of our review. We were unable to include studies in Bengali as there was a paucity of Bengali language literature related to the sociocultural impact of COVID-19. Furthermore, since almost all studies were conducted in Bangladesh, the results and policy-making issues may not be related to developed countries. Since the studies included in the study were from different backgrounds, multiple assessment criteria were used, which could reduce the consistency of assessing.

## Conclusions and Recommendations

This systematic review has analyzed the literature on the sociocultural costs of the COVID-19 pandemic in Bangladesh. This review identifies that long-term disruption in education has led to students’ lack of focus on studies, academic and job uncertainty and threats to normal life. The loss of social communication has made all classes of people lonely and individualistic. Some new social problems have been raised, such as new poor, suicide and violence against women by their partners. Also, the marginal people such as Rohingya faced widespread food insecurity and the upsurge of COVID-19.

We suggest greater attention from the government and community level on initiating integrated actions and policies to combat the problems identified in this review paper. Moreover, to assess the situation further, research-oriented policies and recommendations would best serve the country to tackle the long-term effect of the COVID-19 pandemic. Thus, based on the findings of the review, the following policy recommendations can be taken to improve the sociocultural costs of the long-term COVID-19 crisis in Bangladesh and other nations which are suffering from similar crises:

- Although educational institutions have been reopened, an integrated approach combining both the government and stakeholders, such as teachers or students should be considered to work with the recently suggested United Nations recommendations in order to avoid risks: (a) suppress transmission of the virus and plan thoroughly for school or other institutions reopening, (b) protect education financing and coordinate for impact, (c) build resilient education systems for equitable and sustainable development and (d) reimagine education and accelerate change in teaching and learning (United Nations, 2020);
- The general people should be persuaded to maintain social contact through appropriately distanced in-person visits and digital methods of communication;
- To reduce shadow pandemics- poverty, unemployment and suicide as well as violence against women- necessitating coordinated measures not just from the government and law enforcement authorities, but also a growing awareness among the public, should be taken; and
- The government of Bangladesh and donors should ensure food security to scale up and increase in-kind and voucher food support in the Rohingya camps along with awareness-raising of the spread of COVID-19.
